# Tropical Carathéodory with Matroids

**DOI:** 10.1007/s00454-022-00446-0

**Published:** 2022-11-04

**Authors:** Georg Loho, Raman Sanyal

**Affiliations:** 1 Faculty of Electrical Engineering, Mathematics, and Computer Science, University of Twente, P.O. Box 217, Enschede, Netherlands; 2 FB 12 - Institut für Mathematik, Goethe-Universität Frankfurt, Robert-Mayer-Str. 10, 60325 Frankfurt am Main, Germany

**Keywords:** Colorful Carathéodory Theorem, Matroid Carathéodory Theorem, Tropical convex geometry, 52A35, 05B35, 14T05

## Abstract

Bárány’s colorful generalization of Carathéodory’s Theorem combines geometrical and combinatorial constraints. Kalai–Meshulam (2005) and Holmsen (2016) generalized Bárány’s theorem by replacing color classes with matroid constraints. In this note, we obtain corresponding results in tropical convexity, generalizing the Tropical Colorful Carathéodory Theorem of Gaubert–Meunier (2010). Our proof is inspired by geometric arguments and is reminiscent of matroid intersection. Moreover, we show that the topological approach fails in this setting. We also discuss tropical colorful linear programming and show that it is NP-complete. We end with thoughts and questions on generalizations to polymatroids, anti-matroids as well as examples and matroid simplicial depth.

## Introduction

Imre Bárány’s colorful version of the Carathéodory Theorem is a gem of discrete geometry.

### Theorem 1.1

[7, Thm. 2.1] Let $C_1,\ldots ,C_{d+1}\subset \mathbb {R}^d$ be finite sets of points such that 0 is contained in the convex hull ${\text {conv}}(C_i)$ for all $i=1,\ldots ,d+1$. Then there are $p_i \in C_i$ for $i=1,\ldots ,d+1$ such that $0 \in {\text {conv}}{(p_1,\ldots ,p_{d+1})}$.

The sets $C_i$ are called **color classes** and $\{p_1,\ldots ,p_{d+1}\}$ is called a **colorful simplex**. If $C_1 = \ldots = C_{d+1}$ then this recovers Carathéodory’s original result but Bárány’s theorem exhibits a much more intricate and beautiful interplay of geometry and combinatorics.

There have been many generalizations of Theorem 1.1 mostly weakening the prerequisites for the existence of a colorful simplex containing the origin; see Sect. 2.3 as well as [5, 11, 22]. Kalai and Meshulam [25] gave a different generalization of the Colorful Carathéodory Theorem by interpreting the colorful condition in terms of matroids. A **matroid** $M=(E,\mathcal {I})$ consists of a finite ground set *E* and a non-empty collection of subsets $\mathcal {I}\subseteq 2^E$ satisfying the following conditions: $\mathcal {I}$ is closed under taking subsets and for any $I,J\in \mathcal {I}$ with $|I| < |J|$ there is $e\in J\setminus I$ such that $I\cup e\in \mathcal {I}$. The **rank function** associated to *M* is defined as

$$\begin{aligned} \rho (A):=\max {( |I|\,|\,I \subseteq A,\, I \in \mathcal {I})}. \end{aligned}$$

We refer the reader to [31] for more on matroids.

### Theorem 1.2

[25, Cor. 1.4] Let *M* be a matroid on ground set *E* and $V:E\rightarrow \mathbb {R}^d$. Assume that $0\in {\text {conv}}(V(S))$ for every $S\subseteq E$ with $\rho (S)=\rho (E)$ and $\rho (E\setminus S) \le d$. Then there exists an independent set $T \in \mathcal {I}$ such that $0 \in {\text {conv}}(V(T))$.

Let $E = E_1 \sqcup E_2 \sqcup \cdots \sqcup E_{d+1}$ be a finite set, where $\sqcup$ denotes disjoint union. Define the **partition matroid** *M* with independent sets $I \subseteq E$ if $|I \cap E_i| \le 1$ for all $1 \le i \le d+1$. Then Theorem 1.2 implies Theorem 1.1 with color classes $C_i = V(E_i)$. The rank conditions are a bit tricky to interpret (but quite natural to the proof in [25]). We offer a simple combinatorial interpretation for the case $\rho (E)=d+1$ in Corollary 2.1.

Other geometric setups in which the (Colorful) Carathéodory Theorem holds have been considered, for example tropical geometry. The **(max-)tropical semiring** (or **max-plus-semiring**) is the set $\mathbb {T}_{\max }= \mathbb {R}\cup \{-\infty \}$ together with tropical addition and multiplication given by $a\oplus b := \max (a,b)$ and $a \odot b := a+b$. *Tropical mathematics* is a comparatively young but vibrant area of research that offers new exciting perspectives in virtually all areas of mathematics including algebra, geometry, game theory, and optimization. For tropical convex geometry, one defines the **tropical convex hull** of a finite set $V=\{v^{1},\ldots ,v^{n} \}\subset \mathbb {T}_{\max }^d$ by

1 $$\begin{aligned} {\text {tconv}}(V):=\left\{ \left. \bigoplus _{j=1}^{n} \lambda _j \odot v^{j}\ \right| \ \lambda _j\in \mathbb {T}_{\max }, \;\bigoplus _j \lambda _j = 0\right\} . \end{aligned}$$

We refer the reader to [2, 12] for a thorough introduction to tropical convexity and its connection with classical convexity. Tropical convexity turns out to be useful in gaining new insights into convex optimization; cf. [3]. The combinatorics of tropical convex hulls is further studied in [23]. A first goal of this paper is the study of matroid generalizations of the Colorful Carathéodory Theorem in tropical convex geometry. In Sect. 2.2, we prove the following tropical version of Theorem 1.2.

### Theorem 1.3

Let $M = (E,\mathcal {I})$ be a matroid and $V:E \rightarrow \mathbb {T}_{\max }^d$. Assume that $0 \in {\text {tconv}}(V(S))$ for every $S \subseteq E$ with $\rho (S) = \rho (E)$ and $\rho (E\setminus S) \le d$. Then there exists an independent set $T \in \mathcal {I}$ such that $0 \in {\text {tconv}}(V(T))$.

Gaubert and Meunier [20] proved a tropical Colorful Carathéodory Theorem that, as with Theorem 1.2, is implied by our result. Whereas the tropical Colorful Carathéodory Theorem is a consequence of the pigeonhole principle, our proof is inspired by matroid intersection and the geometric proof of Theorem 1.2 that we give in Sect. 2.1.

Theorem 1.1 was generalized by Holmsen [21] to matroids and oriented matroids. In Sect. 2.3, we discuss the relation to Theorem 1.2 and we prove an analogue for tropical convexity.

The collection of subsets of *E* *not* containing 0 in the convex hull is an abstract simplicial complex, called the *support complex*. The proofs of Kalai–Meshulam and Holmsen build on homological properties of the support complex. In Sect. 2.4 we explain that while the results remain true for tropical convexity, the homological methods fail badly.

In Sect. 3, we study tropical colorful linear programming and we show that just like its classical counterpart, it is NP-complete. We also remark that it is generally not true that tropicalizations of hard problems are hard by considering tropical 0/1-integer linear programming.

The paper closes with Sect. 4 with afterthoughts and questions on generalizations to polymatroids, anti-matroids, examples, and matroid simplicial depth.

## Matroids and Tropical Convexity

### Bases and Cocircuits

We start by giving a simpler combinatorial perspective on Theorem 1.2. The **bases** of a matroid $M=(E,\mathcal {I})$ is the collection $\mathcal {B}\subset \mathcal {I}$ of inclusion-maximal independent sets. The basis exchange property states that for any $B_1,B_2\in \mathcal {B}$ and $e\in B_1\setminus B_2$ there is $f\in B_2\setminus B_1$ such that $(B_1\setminus e)\cup f\in \mathcal {B}$. The **circuits** of *M* are the inclusion-minimal dependent subsets and an element is a **loop** if it forms a singleton circuit. The **dual** of *M* is the matroid $M^*$ with bases $\mathcal {B}^*=\{E\setminus B\,|\,B\in \mathcal {B}\}$. The **cocircuits** $\mathcal {C}^*$ of *M* are the circuits of $M^*$. It is easy to see that each cocircuit of *M* is a minimal transversal of $\mathcal {B}$, that is, any $C\in \mathcal {C}^*$ has a non-empty intersection with every basis $B\in \mathcal {B}$ and it is inclusion-minimal with this property. In fact, for any element *e* in a given basis *B* there is $C\in \mathcal {C}^*$ with $C \cap B=\{e\}$. We call *C* the **fundamental cocircuit** for *B* and *e*.

We give a proof of Theorem 1.2 extending the original arguments of Bárány from [7]; see also the last remark on p. 4 of [21].

#### Proof of Theorem 1.2

If $\rho (E) \le d$, then $\rho (E \setminus S) \le d$ holds for all sets *S* and every basis of *M* verifies the claim. Hence we assume $\rho (E)\ge d+1$. Let *B* be a basis of *M* and let $z \in {\text {conv}}(V(B))$ be the point of smallest Euclidean distance $\delta$ from the origin. If $\delta = 0$ is zero, we are done. If $\delta > 0$, then let *H* the supporting hyperplane to ${\text {conv}}(V(B))$ that contains *z* and that separates ${\text {conv}}(V(B))$ from the origin. There is a subset $I \subset B$ such that $z \in {\text {conv}}(V(I))$ and by the usual Carathéodory Theorem applied to $H \cong \mathbb {R}^{d-1}$, we can assume that $|I| \le d$. Let $H^+$ be the closed halfspace containing ${\text {conv}}(V(B))$ and define $S := \{ e \in E\, |\, V(e) \in H^+ \}$. By construction $B\subseteq S$ and hence $\rho (S) = \rho (B) = \rho (E)$ and $0\notin {\text {conv}}(V(S))$. By assumption, this implies that $\rho (E\setminus S) > d$. Therefore, there is an independent set $J \subseteq E\setminus S$ of size $d+1$ and by the defining property of independent sets, there is $e \in J \setminus I$ such that $I' := I \cup e$ is independent. Extending $I'$ to a basis $B'$, we see that ${\text {conv}}(V(B'))$ contains a point $z'$ of distance $< \delta$ to the origin. As there are only finitely many bases, reiterating the construction with $B'$, we eventually obtain a desired independent set. $\square$

We give a more explicit condition for the (natural) case that the rank is at most $d+1$. For $\rho (E) = d+1$, the condition $\rho (E \setminus S) \le d$ then states that *S* meets every basis, while $\rho (S) = \rho (E)$ means that *S* contains a basis. Note that the set system $\{S \subseteq E\mid \rho (S) = \rho (E), \,\rho (E\setminus S) \le d\}$ is up-wards closed. In particular, for $\rho (E) =d+1$, it is the up-wards closure of $\{B \cup C\mid B\, \text {basis,}\,\, C\, \text {cocircuit of}\, M\}$. This gives the following reformulation of Theorem 1.2.

#### Corollary 2.1

Let *M* be a matroid on the ground set *E* of rank $d+1$ and $V:E \rightarrow \mathbb {R}^d$. Assume that

$$\begin{aligned} 0 \in {\text {conv}}{(V(B \cup C))}\quad \text { for all }\ B \in \mathcal {B}\ \text { and }\ C \in \mathcal {C}^*. \end{aligned}$$

Then there exists a basis $B_0$ such that $0 \in {\text {conv}}(V(B_0))$.

**Fig. 1:**
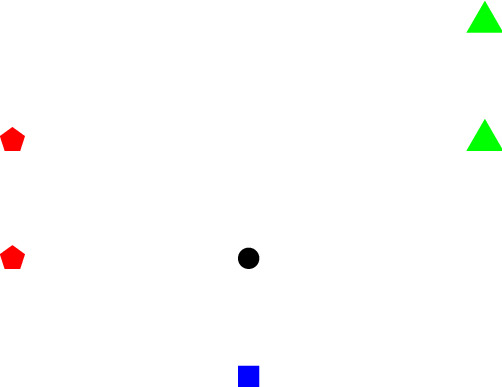
The black circle is contained in the convex hull of any color class with the additional choice of a point from each of the other color classes

If *M* is a partition matroid with $E = E_1 \sqcup \cdots \sqcup E_{d+1}$, then the bases are given by sets *B* with $|B \cap E_i| = 1$. The cocircuits are exactly $\mathcal {C}^*= \{E_1,\ldots ,E_{d+1}\}$. This shows that Theorem 1.2 yields a strengthening of Theorem 1.1: If for all *i* and any choice $p_j \in C_j$ with $j\ne i$ the origin is contained in the convex hull of $C_i \cup \{p_j \,| \,j \ne i\}$, then 0 is contained in the convex hull of a colorful simplex; see Fig. 1.

We focus on the case $\rho (E) = d+1$ to show that we need exactly the structure of a matroid for the proof of Theorem 1.2. For this, recall the following lemma.

#### Lemma 2.2

[14, Lem. 2] Let $M=(E,\mathcal {I})$ be a matroid and $A\in \mathcal {I}$. For $e\in E\setminus A$, we have $A\cup e\in \mathcal {I}$ if and only if there is a cocircuit $C\in \mathcal {C}^*$ with $e \in C$ and $C \cap A = \emptyset$.

#### Proof of Corollary 2.1

Let *B*, *I*, and *H* as in the proof of Theorem 1.2. We can take a fundamental cocircuit *C* for an element in $B\setminus I$. As the origin is contained in ${\text {conv}}{(V(B\cup C))}$, there is an element $c\in C\setminus B$ such that $V(c)\notin H^+$. By Lemma 2.2, $B' = B \setminus C \cup \{c\}$ is a basis. The distance of ${\text {conv}}(V(B'))$ to the origin is strictly smaller than $\delta$. $\square$

We highlight that the property used in the proof exactly characterises matroids among set-systems used to generalize color classes. In terms of general set-systems, the collection of bases of a matroid is a **clutter** (or **Sperner family**): $B \not \subset B'$ for all $B,B' \in \mathcal {B}$. The cocircuits form a **blocker** for $\mathcal {B}$: $C \cap B \ne \emptyset$ for all $C \in \mathcal {C}^*$ and $B \in \mathcal {B}$ and the elements $C\in \mathcal {C}^*$ are inclusion-minimal with this property. Minimality shows that $\mathcal {C}^*$ is a clutter with blocker $\mathcal {B}$. This property of a dual pair of clutters ${\mathcal {C}}$ and ${\mathcal {D}}$, see [19, Cor.], ensures the generalization of the concept of a fundamental cocircuit: for every $H \in {\mathcal {C}}$ and $h \in H$ there is $K \in {\mathcal {D}}$ such that $H \cap K = \{h\}$. The following proposition states that the proof of Theorem 1.2 is *optimal* in the sense that it does not apply to more general clutters.

#### Proposition 2.3

A clutter ${\mathcal {C}}$ is the set of bases of a matroid if and only if, for each *H* in ${\mathcal {C}}$ and *K* in the dual clutter ${\mathcal {D}}$, the set $(H \setminus K) \cup k$ is in ${\mathcal {C}}$ for every $k \in K$.

#### Proof

Sufficiency follows from the definition of cocircuits and Lemma 2.2. For necessity one easily verifies the basis exchange axiom: Let $H_1,H_2\in {\mathcal {C}}$. Fix an element $h\in H_1\setminus H_2$. Let $K \in {\mathcal {D}}$ with $H_1 \cap K=\{h\}$. Since *K* intersects every element of ${\mathcal {C}}$, there is an element $k\in K \cap H_2$. By assumption, $H_1 \setminus \{h\} \cup \{k\}$ is again an element of ${\mathcal {C}}$. This shows the claim. $\square$

#### Remark 2.4

Note that the proofs in this section apply to general ordered fields, such as the field of Puiseux series $\mathbb {R}\{\{t\}\}$. In particular, for tropical configurations $V:E \rightarrow \mathbb {T}_{\max }^d$ that arise as tropicalizations of some $\widehat{V}:E\rightarrow \mathbb {R}\{\{t\}\}_{\ge 0}^d$, Theorem 1.2 implies Theorem 1.3 as well as the results in the next section. Here, the statements of the theorems are interpreted as the containment of a fixed point (and its tropicalization) in certain (tropical) convex hulls, which in general does not have to be the origin. However, actually the containment structure of a tropical convex hull is not necessarily preserved under lifting to $\mathbb {R}\{\{t\}\}^d$. This can be seen from the proof of [16, Prop. 2.1] that each tropical polytope occurs as the image of a polytope over Puiseux series. The lift of a point, which is contained in the tropical convex hull of a finite set *V*(*E*) of points, is not necessarily contained in the convex hull of the lifted points.

### Tropical Greedy Bases

By virtue of tropical convexity, the proof of Theorem 1.3 reduces to a simpler claim about bipartite graphs. To a point $p\in \mathbb {T}_{\max }^d$, we associate a decomposition of $\mathbb {T}_{\max }^d$ into $d+1$ affine sectors. For $i \in [d+1] := \{1,\ldots ,d+1\}$, we define the *i* **-th affine sector** as

2 $$\begin{aligned} \begin{aligned} S_i(p)&:=\{z \in \mathbb {T}_{\max }^d\mid z_i + p_k \ge z_k + p_i \text { for } 1 \le k \le d+1\} \\&=\Bigl \{z \in \mathbb {T}_{\max }^d\;\big |\;z_i - p_i = \max _{k \in [d+1]}(z_k - p_k)\Bigr \} \end{aligned} \end{aligned}$$

where we set $p_{d+1} := z_{d+1} := 0$. For a point $v \in \mathbb {T}_{\max }^d$ we set

$$\begin{aligned} \mathcal {N}_p(v):=\{i \in [d+1]\mid v \in S_i(p)\}. \end{aligned}$$

The **covector graph** $G_V(p)$ of *p* with respect to *V* is the bipartite graph on $V \sqcup [d+1]$ with edges (*v*, *i*) for $i \in \mathcal {N}_p(v)$. If the covector graph *G* is fixed, we will use $\mathcal {N}_G(v) \subseteq [d+1]$ for neighborhood of $v \in V$.

The next lemma characterizes the containment of a point with finite coordinates; we omit the version for points with $-\infty$ entries as we do not need it in the following.

#### Lemma 2.5

[15, Prop. 9], [24, Lem. 28] The point $p \in \mathbb {R}^d$ is contained in the tropical convex hull of $v^1,\ldots , v^n$ if and only if no node of $[d+1]$ is isolated in $G_V(p)$.

Note that, by construction, no node of *V* is isolated in $G_V(p)$.

#### Proof of Theorem 1.3

Let $G = G_V(0)$ be the covector graph for $p = 0$ and $V = V(E)$. In analogy to our proof of Theorem 1.2, we start with an arbitrary basis *B* of *M*. If 0 is not contained in the tropical convex hull of *V*(*B*), the neighborhood $\mathcal {N}_G(B) = \bigcup _{e \in B} \mathcal {N}_G(e)$ in *G* is a strict subset of $[d+1]$. Using the tropical colorful Carathéodory theorem (i.e., the pigeonhole principle applied to the covector graph *G*), there is a subset *I* of *B* of cardinality at most *d* with $\mathcal {N}_G(I) = \mathcal {N}_G(B)$.

Let $S \subseteq E$ be maximal with $\mathcal {N}_G(S) \subseteq \mathcal {N}_G(B)$. Clearly we have $B \subseteq S$, which implies $\rho (S) = \rho (E)$. As $\mathcal {N}_G(S)$ is a strict subset of $[d+1]$, we have $0 \not \in {\text {tconv}}(V(S))$. Hence, the assumption yields $\rho (E \setminus S) > d$. Therefore, there is an independent subset *J* of $E \setminus S$ of rank $d+1$. Using matroid augmentation, there is an element $e \in J \setminus I$ such that $I \cup e$ is independent and can be extended to a basis $B'$ of *M*. By construction, the neighbourhood $\mathcal {N}_G(B')$ strictly contains $\mathcal {N}_G(B)$. As its cardinality is at most $d+1$, iterating this construction yields a desired set in at most $d+1$ steps. $\square$

### Unions of Color Classes

Another strengthening of the Colorful Carathéodory Theorem is obtained by considering unions of color classes.

#### Theorem 2.6

[5, Thm. 1], [22, Thm. 5] Let $C_1,\ldots ,C_{d+1} \subset \mathbb {R}^d$ be finite sets of points such that 0 is contained in the convex hull ${\text {conv}}{(C_i \cup C_j)}$ for all $i \ne j$. Then there are $p_i \in C_i$ for $i=1,\ldots ,d+1$ such that $0 \in {\text {conv}}{(p_1,\ldots ,p_{d+1})}$.

A generalization of this result, which also replaces convex hulls in Euclidean space by oriented matroids is due to Holmsen [21].

#### Theorem 2.7

[21, Thm. 1.2] Let *M* be a matroid with rank function $\rho$ and let ${\mathcal {O}}$ be an oriented matroid of rank *d*, both defined on the same ground set *V* and satisfying $\rho (V) = d+1$. If every subset $S \subset V$ such that $\rho (V\setminus S) \le d-1$ contains a positive circuit of ${\mathcal {O}}$, then there exists a positive circuit of ${\mathcal {O}}$ contained in an independent set of *M*.

**Fig. 2:**
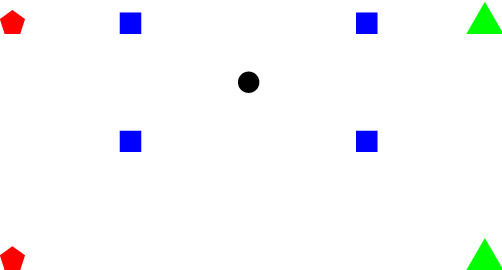
A configuration fulfilling the prerequisites of Theorem 2.6 but not of Theorem 1.2/Corollary 2.1

Note that the statement also requires $\rho (V) = d+1$ as the simplified version of Theorem 1.2 given in Corollary 2.1. Furthermore, Theorem 2.7 is not a strengthening of Theorem 1.2! Fig. 2 illustrates this.

Observe that the union of two cocircuits in a partition matroid of rank $d+1$ is a minimal set whose complement has rank $d-1$. This yields the matroid generalization of Theorem 2.6.

#### Corollary 2.8

Let *M* be a matroid on the ground set *E* and $V:E \rightarrow \mathbb {R}^d$. If the origin is contained in the convex hull ${\text {conv}}(V(S))$ for each set *S* that meets every basis in at least two elements, then there is a basis $B_0$ such that $0 \in {\text {conv}}(V(B_0))$.

For tropical convexity, we obtain a matroid generalization of the union of two color classes, which is slightly weaker than the condition in Corollary 2.8.

#### Theorem 2.9

Let $M = (E,\mathcal {I})$ be a matroid of rank $d+1$ and $V:E \rightarrow \mathbb {T}_{\max }^d$. Assume that

$$\begin{aligned} 0 \in {\text {tconv}}{(V(C \cup D))} \quad \text { for any two distinct cocircuits }\ C, D \text { of } M. \end{aligned}$$

Then there exists a basis $B_0$ such that $0 \in {\text {tconv}}(V(B_0))$.

#### Proof

Again using Lemma 2.5, we translate the claim into a problem on the covector graph $G_V(0)$ of 0 with respect to *V*(*E*). Let *B* be a basis whose neighborhood $\mathcal {N}_G(B)$ does not cover $[d+1]$. We pick a surjective map $\phi$ from *B* to $\mathcal {N}_G(B)$. Since $|B| = d+1>|\mathcal {N}_G(B)|$, there are two elements $p,q \in B$ with $\phi (p) = \phi (q)$. Let $C_p, C_q$ be cocircuits with $C_p \cap B = \{p\}$ and $C_q \cap B =\{q\}$. As $\mathcal {N}_G(C_p \cup C_q) = [d+1]$, there is an element $r \in C_p\cup C_q$ with $\mathcal {N}_G(r) \setminus \mathcal {N}_G(B) \ne \emptyset$. We can assume that $r \in C_p$. We conclude that $B \setminus \{p\} \cup \{r\}$ is a basis and covers more elements than *B*. $\square$

### Support Complexes

The proofs in [25] and [21] rely on topological methods. The **support complex** (or **avoiding complex** in [1]) of $V:E \rightarrow \mathbb {R}^d$ is the abstract simplicial complex

$$\begin{aligned} \Delta _V:=\{ \sigma \subseteq E\mid 0 \notin {\text {conv}}(V(\sigma ))\}\subseteq 2^E. \end{aligned}$$

The structure of support complexes is special. In both papers, the proofs make use of the fact that the complexes are *d*-collapsible or *near d-Leray*, which is a condition on the vanishing of the homology of links of $\Delta _V$.

In the tropical setting, the **tropical support complex** can be read off the covector graph $G_V(0)$

$$\begin{aligned} \Delta _V=\{\sigma \subseteq E\mid \mathcal {N}_G(\sigma ) \ne [d+1] \}. \end{aligned}$$

The following result shows that the topological approach does not work in the tropical setting.

#### Proposition 2.10

For any simplicial complex $\Delta \subseteq 2^{E}$ with *m* facets, there is a configuration $V:E \rightarrow \mathbb {T}_{\max }^{m-1}$ such that $\Delta = \Delta _V$.

#### Proof

Let $\sigma _1,\ldots ,\sigma _m \subseteq E$ be the facets of $\Delta$. Consider the bipartite graph *G* on $E \sqcup [m]$ such that the neighborhood of $i \in [m]$ is precisely $E \setminus \sigma _i$. This is realized as covector graph $G_V(0)$ by the point configuration $V:E \rightarrow \mathbb {T}_{\max }^{m-1}$ such that for $e\in E$ we have

$$\begin{aligned} V(e)_i={\left\{ \begin{array}{ll}-\infty &{} \text {if } e \in \sigma _i,\\ 0 &{} \text {if } e \notin \sigma _i.\end{array}\right. } \end{aligned}$$

The inclusion-maximal subsets $\sigma \subseteq E$ whose neighborhoods fail to cover [*m*] miss some $i \in [m]$ and hence the facets of $\Delta _V$ are exactly $\sigma _1,\ldots ,\sigma _m$. $\square$

## Tropicalized Colorful Linear Programming

Colorful linear programming was introduced in [8] as an algorithmic framework originating from the Colorful Carathéodory Theorem [7]: Given finite sets $C_1,\ldots ,C_k \subset \mathbb {R}^d$ and $b \in \mathbb {R}^d$, decide if there is $p_i \in C_i$ for $i=1,\ldots ,k$ such that $b \in {\text {conv}}{(p_1,\ldots ,p_k)}$. If $k=d+1$ and $C_1 = C_2 = \ldots = C_{d+1}$, then this boils down to the question if a given point is contained in the convex hull of a finite set of points. Writing the conic version of this gives rise to the feasibility problem for a linear program in equality form with non-negative variables.

Many interesting problems can be modeled as colorful linear programs and it continues to be a topic of active research; see [29]. In this section, we consider tropical colorful linear programming and we show that it is NP-complete.

For classical colorful linear programming, NP-completeness was already shown by Bárány and Onn [8]. It might therefore not come as a surprise that the same holds true in the tropical setting. This intuition, however, is misguided. To set the stage, we start with a discussion on tropical integer linear programming that, unlike its classical counterpart, is in the complexity class *P*.

### Tropicalized Integer Programming

The 3-dimensional matching problem is one of the classical NP-complete problems whose hardness was already established by Karp in his seminal paper [26]. In the exact cover version, we are given three disjoint sets *A*, *B*, *C* of the same finite cardinality *k*. Given a 3-uniform 3-partite hypergraph ${\mathcal {H}}$ on $A\cup B\cup C$, namely a collection of hyperedges ${\mathcal {H}}$, where $|h\cap A|=|h\cap B|=|h\cap C|=1$ for each $h\in {\mathcal {H}}$, then a $\textbf{3}$ **-dimensional matching** is a subset $M \subseteq {\mathcal {H}}$ such that

$$\begin{aligned} \bigcup _{w \in M} w = A \cup B \cup C \qquad \text { and } \qquad u \cap v =\emptyset \quad \text {for all }\ u,v \in M,\ u \ne v. \end{aligned}$$

The algorithmic problem can be formulated as a 0/1-integer program where not only the variables but also the coefficients are from $\{0,1\}$. We introduce a variable $x_h$ for each hyperedge $h \in {\mathcal {H}}$. The existence of a 3-dimensional matching is equivalent to the feasibility of the system

3 $$\begin{aligned} \begin{aligned} \sum _{h \ni w} x_h = 1&\quad \text { for all } w \in A \cup B \cup C, \\ x_h \in \{0,1\}&\quad \text { for all } h \in {\mathcal {H}}. \end{aligned} \end{aligned}$$

This demonstrates that already this restricted version of integer linear programming is NP-complete.

Butkovič [13] proposed a version of tropical integer linear programming based on the lattice points ${\mathbb {Z}} \subset {\mathbb {R}}$. However, unlike for classical integer programming, this notion of lattice points does not allow for more modeling power as a feasible tropical linear program with integer coefficients anyway always has an integer solution. A different approach comes from observing that $-\infty$ and 0 are the tropical additive and multiplicative neutral elements, respectively. The corresponding class of tropical 0/1-integer programs are systems of inequalities where the coefficients are restricted to $-\infty$ or 0, the neutral elements for tropical addition and multiplication. For $J \subseteq [n]$ the tropical analog of the equations in (3) is

$$\begin{aligned} \bigoplus _{k \in J}\,x_k=0 \end{aligned}$$

with the additional condition $x_k \in \{-\infty ,0\}$. More generally, we say that a **tropical** $\{-\infty ,0\}$**-integer linear program** is the feasibility problem with solutions in $\{-\infty ,0\}^n$ for a system with inequalities of the form

4 $$\begin{aligned} \max _{i \in I} x_i\le \max _{k \in J} x_k \end{aligned}$$

with $I, J \subseteq [n]$ such that $I \cap J = \emptyset$, where potentially some of the variables are fixed to 0 or $-\infty$ in advance. An inequality of the form (4) is equivalent to

$$\begin{aligned} x_i\le \max _{k \in J} x_k\quad \text { for all }\ i \in I. \end{aligned}$$

Associating a Boolean variable $z_i \in \{\bot ,\top \}$ for each $x_i$ with $z_i = \bot$ if and only if $x_i = -\infty$, we can interpret such a tropical inequality as a Boolean formula

$$\begin{aligned} z_i \Rightarrow \bigvee _{k \in J} z_k\quad \text { or, equivalently, } \quad \lnot z_i \vee \bigvee _{k \in J} z_k. \end{aligned}$$

This is a dual Horn clause. A system of equations (4) comprises a conjunction of these clauses. Hence, the feasibility of such a tropical $\{-\infty ,0\}$-integer linear program is equivalent to the satisfiability problem for Horn formulae. This is known to be linear-time solvable [18] but also P-complete as illustrated in the recent survey [6].

We summarize the preceding discussion in the following statement which is in contrast to the observation that classical integer linear programming is hard even with coefficients from $\{0,1\}$.

#### Theorem 3.1

Tropical $\{-\infty ,0\}$-integer linear programs can be solved in polynomial time.

A slightly different point of view is taken in [30]. They consider scheduling with AND-OR networks which in its most general form is equivalent to tropical linear programming. The problem, which they consider in [30, Thm. 3.1], is essentially the same as here. The unifying language of hypergraph reachability was already used in [4] to express a similar problem for tropical polyhedra. It can also be seen from the point of constraint satisfaction problems; see [10].

#### Example 3.2

The direct ‘tropicalization’ of (3) results in

$$\begin{aligned} A \odot x=\varvec{0}\quad \text { with }\ x \in \{-\infty ,0\}^{{\mathcal {H}}}, \end{aligned}$$

where $A \in \{-\infty ,0\}^{U \times {\mathcal {H}}}$ is the hyperedge-node-incidence matrix of the hypergraph ${\mathcal {H}}$. The idempotency of $\max$ significantly weakens constraints. While the equations arising in (3) enforce that exactly one part of the tripartition $A \cup B \cup C$ is hit, the tropical equations only encode the covering condition. In particular, already the choice $x = \varvec{0}$ serves as solution. As stated in the discussion after [12, Cor. 3.1.3], this problem becomes NP-complete only by imposing an additional minimality condition.

### Tropicalized Colorful Linear Programming

The tropical version of the ‘positive dependence version’ treated in [8, Sect. 5] is the following problem. Let $A \in \mathbb {T}_{\max }^{d \times n}$ and $C_1 \sqcup C_2 \sqcup \cdots \sqcup C_r = [n]$ a partition. Find $x \in \mathbb {T}_{\max }^{n}$ such that

5 $$\begin{aligned} A \odot x=\varvec{0}\ \quad \text { and }\ \quad |{{{\,\textrm{supp}\,}}(x)\cap C_i}| = 1\ \text { for all } i =1,\ldots ,r. \end{aligned}$$

Here ${{\,\textrm{supp}\,}}(x) = \{ i\,|\,x_i \ne -\infty \}$. Note that we use a homogeneous formulation of this dependence relation omitting the condition that the components of *x* tropically add up to tropical one. The following is a tropical analogue to [8, Thm. 5.1].

#### Theorem 3.3

Tropical colorful linear programming is NP-complete.

Note that a similar argument on NP-completeness of *unique solvability* already occurs in [12, Sect. 3.1].

#### Proof

It is clear that the feasibility of some $x \in \mathbb {T}_{\max }^n$ can be checked in polynomial time and the NP-hardness is the nontrivial part. To prove hardness, we use a similar construction as in Example 3.2. Let ${\mathcal {H}}$ be 3-uniform 3-partite hypergraph on nodes $U = A \sqcup B \sqcup C$ with $|A| = |B|= |C| = k$. For every edge $h = \{a,b,c\} \in {\mathcal {H}}$, define $v_h \in \mathbb {T}_{\max }^{U}$ by

$$\begin{aligned} (v_h)_r={\left\{ \begin{array}{ll}0 &{} \text { if } r \in \{a,b,c\}, \\ infty &{} \text { otherwise.}\end{array}\right. } \end{aligned}$$

For $V=\{v_h\,|\,h \in {\mathcal {H}}\}$, we use (2) to see that the covector graph $G_V(0)$ for the point $p = 0$ is the bipartite graph on nodes ${\mathcal {H}}\sqcup U$ with edges (*h*, *a*), (*h*, *b*), and (*h*, *c*) for every $h = \{a,b,c\} \in {\mathcal {H}}$. In particular, every $h \in {\mathcal {H}}$ has three incident edges in $G_V(0)$.

Now define color classes $C_a = \{ (a,b,c) \in {\mathcal {H}}\mid b \in B,\,c \in C\}$ for $a \in A$ and let $x \in \mathbb {T}_{\max }^{\mathcal {H}}$ such that

$$\begin{aligned} \bigoplus _{h \in {\mathcal {H}}}\, v_h \odot x_h=0 \end{aligned}$$

with $|{{{\,\textrm{supp}\,}}(x)\cap C_a}|=1$ for all $a\in A$. Then Lemma 2.5 yields that $M = {{\,\textrm{supp}\,}}(x)$ covers *A*, *B*, and *C*. Since $|M| = |A| = |B| = |C|$, it follows that *M* is in fact a matching. $\square$

#### Remark 3.4

A potential application of the above described version of tropical colorful linear programming to *constrained scheduling problems* arises in the following way. By replacing everything with its negative, we can switch from $\max$ to $\min$; in particular, this involves replacing $-\infty$ by $+\infty$. Consider again (5). We want to finish *d* jobs at fixed termination times (the vector $\varvec{0}$). They are based on fixed processing times (the entries of *A*). The columns of *A* correspond to *n* different ways to finish a job. For each job (resp. row), at least one way has to be finished at the termination time. The color classes can be used to model additional production constraints. For each way of finishing a job (resp. the columns of *A*), only one of the potential approaches in a color class $C_i$ for $i \in [r]$ can be chosen. This restriction could be imposed if all approaches in a color class are executed on the same unit.

## Afterthoughts and Questions

### Polymatroid Carathéodory Theorem

Theorems 1.2 and 1.3 can be phrased completely in terms of the rank function $\rho :2^E\rightarrow \mathbb {Z}_{\ge 0}$ as independent sets satisfy $\rho (I) = |I|$. In this form, Theorem 1.3 is reminiscent of Rado’s generalization of Hall’s marriage theorem. Welsh [34] further generalized Rado’s result to polymatroids. A **polymatroid** is a function $f:2^E\rightarrow \mathbb {Z}_{\ge 0}$ that is monotone with respect to inclusion and submodular $f(A \cup B) + f(A \cap B) \le f(A) +f(B)$ for all $A,B \subseteq E$. See [31, Sect. 11.2] for a discussion of all three results.

#### Theorem 4.1

Let $(A_j)_{j \in J}$ be a family of non-empty subsets of *E* and let $f:2^E \rightarrow \mathbb {Z}_{\ge 0}$ be a polymatroid. Then there is a choice $e_j \in A_j$ for $j \in J$ such that

$$\begin{aligned} f(\{ e_j\,|\,j \in K\})\ge |K| \quad \ \text { for all }K \subseteq J \end{aligned}$$

if and only if

$$\begin{aligned} f(A(K))\ge |K| \quad \ \text { for all } K \subseteq J, \end{aligned}$$

where $$A(K) = \bigcup _{j \in K} A_j$$.

For $f = \rho$, this is Rado’s result. For $f(A) = |A|$, this gives Hall’s marriage theorem.

#### Question 1

Can Theorems 1.2 and 1.3 be further generalized to polymatroids?

A first attempt for a very restricted class of polymatroids was done in [33]. While Theorem 4.1 is actually a consequence of Edmond’s matroid intersection, we also get another connection with the interplay of two matroids arising from the consideration of generic configurations. As we saw in Sect. 2.2, the containment property for the tropical convex hull translated to a covering property in the associated covector graph.

For the case that 0 is in generic position with respect to *V*, we can give a necessary and sufficient condition. The genericity means that in the covector graph *G* each node in *E* has degree 1. Equivalently, the set system $(\mathcal {N}_G(i))_{i\in [d+1]}$ forms a partition matroid on *E*.

The point *p* is contained in the tropical convex hull of *V*(*I*) for an independent set *I* of *M* if and only if *I* is a spanning set of the partition matroid $(\mathcal {N}_G(i))_{i\in [d+1]}$.

This means that $(\mathcal {N}_G(i))_{i\in [d+1]}$ contains a transversal $(x_i)_{i\in [d+1]} \subseteq E$ such that $\{x_i\,|\,i \in [d+1]\}$ is independent in *M*. Equivalently, there is a transversal $(x_i)_{i \in [d+1]}$ such that

$$\begin{aligned} \rho (\{x_j \,|\,j \in J\}) \ge |J| \quad \ \text { for all }J\subseteq [d]. \end{aligned}$$

Indeed, the inequality has to be fulfilled with equality. Now, Theorem 4.1 yields the following equivalence.

#### Proposition 4.2

If 0 is in generic position with respect to *V* then 0 is in the tropical convex hull of an independent set if and only if

$$\begin{aligned} \rho \left( \,\bigcup _{j \in J} \mathcal {N}_G(j)\right) \ge |J| \quad \ \text { for all }J \subseteq [d]. \end{aligned}$$

### Convex Geometries

A **convex geometry** or **anti-matroid** is a pair $(E,\tau )$ where $\tau :2^E \rightarrow 2^E$ is a hull operator that satisfies the *anti-exchange axiom*: for $A \subseteq E$ and $x,y \notin \tau (A)$ with $x \ne y$

$$\begin{aligned} x \in \tau ( A \cup y) \ \Longrightarrow \ y \notin \tau ( A \cup x). \end{aligned}$$

For example, if $E \subset \mathbb {R}^d$ is a finite set, then $\tau (A) := {\text {conv}}(A)\cap E$ defines a convex geometry. The definition of tropical convex hulls and Lemma 2.5 implies the following.

#### Proposition 4.3

Let $E \subseteq \mathbb {T}_{\max }^d$ be a finite set. Then $\tau (A) := {\text {tconv}}(A)\cap E$ defines a convex geometry.

This naturally raises the following question:

#### Question 2

Is there a generalization of Theorems 1.2 and 2.7 for convex geometries?

The first question to be answered here is what should take place of the dimension? A natural choice is the **Carathéodory number** of $(E,\tau )$, that is, the smallest number $c = c(E,\tau )$ such that for any $A \subseteq E$ and $e \in \tau (A)$, there is $A' \subseteq A$ with $|A'| \le c$ and $p \in \tau (A')$. For the convex geometries above and *E* in general position, the Carathéodory number is $d+1$ and suggest the right generalization. Unfortunately, the following example shows that the Carathéodory number is not a suitable replacement.

#### Example 4.4

Let $G=(V,E)$ be a connected graph with distinguished node $r\in V$. A subset $F\subseteq E$ is **feasible** if the edge-induced subgraph *G*[*F*] is connected and contains *r*. A set $C \subseteq E$ is **convex** if $E \setminus C$ is feasible. If we define $\tau (A) =\bigcap \,\{ C\,|\,A \subset C, \,C \text { convex}\}$, then $(E,\tau )$ is a convex geometry; see [27, Exam. 2.10].

**Fig. 3:**
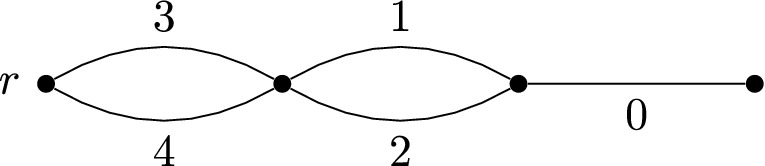
Graph for Example 4.4

Consider the convex geometry for the graph in Fig. 3. It can be checked that the Carathéodory number for this convex geometry is 2. Indeed, $\{0,1,2\} \subset \tau (\{3,4\})$, $0 \in \tau (\{1,2\})$ and 3, 4 are the extreme points of $\tau (E)$. Let $C_1 = \{1,2\}$ and $C_2 = \{3,4\}$ be color classes. Then $0 \in \tau (C_1) \cap \tau (C_2)$ but $0 \notin \tau (\{p_1, p_2\})$ for any choice $p_i \in C_i$.

The Radon number for this example is 3. It would be interesting to know if the Radon number is a suitable replacement for dimension.

Let us finally note that the topological approach of [25] and [21] does not apply. For a convex geometry $(E,\tau )$ and $p \in E$, one defines the support complex $\Delta _p := \{ A \subseteq E\mid p\notin \tau (A)\}$. However, Propositions 4.3 and 2.10 imply that any simplicial complex is the support complex of a convex geometry.

### Examples and Matroid Simplicial Depth

It turns out that, as compared to the Colorful Carathéodory Theorem 1.1, it is quite difficult to construct non-trivial instances for Theorem 1.2 where *M* is not the partition matroid.

Our reformulation of Theorem 1.2 in Corollary 2.1 shows that the conclusion is trivial for $\mathcal {C}^*\cap \mathcal {I}\ne \emptyset$. This is equivalent to the statement that for every basis $B \in \mathcal {B}$ there is a basis $B' \in \mathcal {B}$ with $B\cap B'=\emptyset$. For example, if *M* is a graphical matroid associated to a graph *G*, then for every node $v \in V(G)$, the edges incident to *v* give a cocircuit, which can be completed to a basis. So graphical matroids do not give non-trivial instances for Theorem 1.2 independent of the choice of $V:E \rightarrow \mathbb {R}^d$.

#### Question 3

Find a natural class of instances of Theorem 1.2.

Deza et al. [17] introduced the **colorful simplicial depth** of a colorful configuration $C_1,\ldots ,C_{d+1} \subset \mathbb {R}^{d}$ as the maximal number of colorful simplices intersecting in a point. They conjectured upper and lower bounds for color configurations in $\mathbb {R}^d$ that was shown to be true; see [1, 32]. For any fixed matroid *M*, one can introduce the analogous notion of *matroid simplicial depth*.

#### Question 4

What are lower and upper bounds on the matroid simplicial depth?

The techniques in [1] give an approach: For $M =(E,\mathcal {I})$ and $V:E \rightarrow \mathbb {R}^d$, define the **M-avoiding complex**

$$\begin{aligned} \Delta _M:=\{ I \in \mathcal {I}\mid 0 \notin {\text {conv}}(V(I)) \}=\Delta _V\cap \mathcal {I}. \end{aligned}$$

This is a subcomplex of the independence complex $\mathcal {I}$ and the proof of [1, Lem. 2.2] yields

#### Proposition 4.5

Let *M* be a matroid and $V:E \rightarrow \mathbb {R}^d$. Then the matroid simplicial depth is bounded from above by

$$\begin{aligned} (-1)^{|E| - \rho (E)}\mu (M^*) + \tilde{\beta }_{d-1}(\Delta _M), \end{aligned}$$

where $\mu (M^*)$ is the Möbius invariant of the dual matroid and $\tilde{\beta }_{d-1}$ is the reduced Betti number (over $\mathbb {Z}_2$) of $\Delta _M$.

#### Proof

The proof of [1, Lem. 2.2] verbatimly carries over. The missing ingredient is the Euler characteristic of $\mathcal {I}$. This was determined in [9, Prop. 7.4.7] to be $(-1)^{|E| - \rho (E)} \mu (M^*)$. $\square$

To show that this upper bound is tight for the partition matroid it is shown that any configuration $V:E \rightarrow \mathbb {R}^d$ can be transformed to a natural configuration that attains the bound and such that in every step the colorful simplicial depth does not decrease.

### Signed Tropical Colorful Linear Programming

The notion of tropical convexity considered here is restricted to the tropical non-negative numbers, as $x \ge -\infty$ for all $x\in \mathbb {T}_{\max }$. Recently in [28], the extended concept of signed tropical convexity has been developed. Instead of considering only points with coordinates in $\mathbb {T}_{\max }$, one glues the non-negative numbers $\mathbb {T}_{\max }$ with a negative copy $\ominus \mathbb {T}_{\max }$ at $-\infty$ arriving at ${\mathbb {T}}_{\pm }= \mathbb {R}\cup \{-\infty \} \cup \ominus \mathbb {R}$.

#### Question 5

Can Theorems 1.3 and 2.9 be generalized to signed tropical convexity?

It was shown that deciding the containment of the origin $(-\infty ,\ldots ,-\infty )$ in the convex hull of finitely many point in ${\mathbb {T}}_{\pm }^d$ is equivalent to tropical linear programming.

We introduced tropical colorful linear programming in Sect. 3.2 where no variable occurs on the right hand side in (5). Considering the containment for $(-\infty ,\ldots ,-\infty )$ instead of $(0,\ldots ,0)$ in the sense of signed tropical convexity yields a generalization of the two-sided version of tropical linear programming and our one-sided version of tropical colorful linear programming.

#### Corollary 4.6

Signed tropical colorful linear programming is NP-complete.

It was shown in [28] that the most general version of signed tropical linear programming is NP-complete, while a tamer version with non-negative variables is in $NP \cap co-NP$ as it is equivalent to mean payoff games; see [30].

The modeling power of non-tropical colorful linear programming was greatly demonstrated in [29].

#### Question 6

Which problems can be modeled by signed tropical colorful linear programming?
